# Special Issue: Hypoxia-Inducible Factors: Regulation and Therapeutic Potential

**DOI:** 10.3390/biomedicines9121768

**Published:** 2021-11-25

**Authors:** Kiichi Hirota

**Affiliations:** Department of Human Stress Response Science, Institute of Biomedical Science, Kansai Medical University, Hirakata 573-1010, Osaka, Japan; khirota-kyt@umin.ac.jp; Tel.: +81-72-804-2526

Oxygen (O_2_) is an essential molecule [1] in the production of adenosine triphosphate (ATP) in cells, and a lack of energy due to O_2_ deficiency makes the maintenance of biological functions and human life improbable. Since oxygen functions as the final electron acceptor in the series of ATP synthesis reactions in conjunction with oxidative phosphorylation in mitochondria, its deficiency causes the oxidation of a series of coenzymes such as nicotinamide and flavin adenine dinucleotide and the reduction in oxygen molecules to water molecules (H_2_O). Persistent deficiency has been believed to cause to the loss of biological functions, even resulting in death. This classical view of oxygen has been completely revised over the last 20 years. Mammals do not have a mechanism for biosynthesizing oxygen in their bodies. In higher organisms such as vertebrates, which possess many organs, oxygen in the body is always “scarce,”; therefore, the dominant view is that organisms have evolved mechanisms to respond to the lack of this essential molecule (hypoxia), and actively use it to maintain body integrity [2,3].

Anatomically complex, higher multicellular organisms are equipped with specialized mechanisms to enable all cells to obtain sufficient oxygen. The respiratory system consists of lungs, which provide oxygen to be transferred to hemoglobin in red blood cells, the diaphragm, other respiratory support muscles, and neuroepithelial cells that sense the partial pressure of oxygen. The cardiovascular system consists of red blood cells, oxygen-carrying medium, the heart, the transport engine, blood vessels, and transport channels. The proper development and preservation of these systems require the harmonious expression of thousands of genes. The transcription factor responsible for such gene expression is hypoxia-inducible factor 1 (HIF-1) [3].

In the late 1980s, a team at Johns Hopkins University in Baltimore, USA, searched for an intracellular factor involved in the hypoxia-induced expression of erythropoietin (Epo) and isolated the complementary DNA (cDNA) of a transcription factor in 1995 [4]. This transcription factor was named HIF-1 [5,6,7]. A closely related gene, hypoxia-inducible factor 2α (*HIF2A*) or endothelial PAS domain protein 1 (*EPAS1*), was identified and cloned in 1997, followed by hypoxia inducible factor 3α (*HIF3A*) in 1998 [8]. A series of genes, including those coding for various glycolytic enzymes, glucose transport proteins, vascular endothelial growth factor, and hematopoietic factor Epo, are regulated by HIFs at the transcriptional level. Therefore, HIFs play a role in the activation of “hypoxia-inducible” genes, and we can refer to the term “HIF” as “Highly Involved Factor” [9].

In 2019, the Nobel Prize in Physiology or Medicine was awarded to three researchers for outstanding achievements in this field [10,11,12].

In this Special Issue, we invited research and review papers in various areas of oxygen biology research that focused on the fundamental understanding of HIF signaling pathways and related gene expression profiling, as well as pharmacogenomic biomarkers, molecular targets driving the regulation of human physiology and pathophysiology, and validation in animal models. As a result, we published six original papers and three review articles in this Special Issue [1,13,14,15,16,17,18,19,20].

Changes in gene expression in response to hypoxic stimuli were studied, with the discovery of hypoxia response systems represented by HIFs. Bono et al. performed a meta-analysis of RNA-sequencing data from hypoxic transcriptomes archived in public databases. In addition, meta-analyzed hypoxic transcriptome data were integrated with public chromatin immunoprecipitation-sequencing data on the known human HIFs, HIF-1, and HIF-2, to provide insights into hypoxia-responsive pathways involving direct transcription factor binding. This study serves as a useful resource for hypoxia research [13]. However, Bono and Ono hypothesized that some hypoxia-responsive genes might not have yet been discovered, hiding behind famous genes. Furthermore, they searched for novel hypoxia-responsive genes in a data-driven manner by utilizing biodigital transformation [19], where data accumulated in a public database (DB) were used for research. They constructed a meta-analysis method to evaluate variations in the expression levels of all genes collected from public databases, before and after hypoxic stimulation, and discovered several novel hypoxia-responsive genes using a multi-omics analysis that integrated information on all genes in the article database. They also created a data-driven meta-analysis method to evaluate alterations in the expression information (transcriptome) of all genes with and without hypoxic stimulation collected from public databases. Thus, they discovered several novel hypoxia-responsive genes through a multi-omics analysis that integrated the collected transcriptome information with genes published in all papers (bibliome) in the public DB.

The secretome is expected to be useful in biomarker discovery because it contains abundant proteins and fragments of membrane proteins secreted and released by cancer cells, unlike blood samples, which do not contain secretions from various tissues.

The term “secretome” is a general term that includes not only soluble proteins released from cells via the endoplasmic reticulum (ER)-Golgi pathway, but also extracellular matrix proteins and cleaved fragments of membrane proteins. Moog et al. have shown that the properties of the secretome are affected by hypoxia in a series of studies [16,17,18].

It has been reported that HIF-1 regulates the induction of matrix metalloproteinases (MMPs). Wei et al. found that xanthine oxidase-derived reactive oxygen species (ROS) induced MMP-3, MMP-10, and MMP-13 in mouse macrophages upon hypoxic exposure. Induction was not inhibited by HIF-1α-deficient macrophages, but was HIF-1-independent. Additionally, induction was found to be inhibited by febuxostat, a xanthine oxidase inhibitor. Febuxostat administration is a potential therapeutic option for disease management in atherosclerotic patients [20].

The role of oxygen metabolism in the regulation of inflammation and immune responses has attracted attention. Humoral factors, including cytokines and chemokines, and physical stimuli such as heat and tissue breakdown induce ROS and generate oxidative stress, while impaired blood vessels, impaired blood flow, increased oxygen consumption by infiltrating cells, and impaired oxygen diffusion due to edema reducing the oxygen concentration in tissues. Inflammatory cells, which work “in the field” of inflammation, adapt to these changes in the oxygen environment to maintain cellular functions and contribute to biological defense and homeostasis [21,22]. Chen and Gaber summarized the effects of physiological and pathophysiological hypoxia on innate and adaptive immune activity. We provide an overview on the control of immune response by cellular hypoxia-induced pathways, with a focus on the role of HIFs, and discuss the opportunity to target hypoxia-sensitive pathways for the treatment of cancer and autoimmunity [14].

HIF plays an essential role in this process; it is a transcription factor that mediates Epo induction at the transcriptional level under hypoxic conditions. In 2001, cDNA cloning of dioxygenases acting on prolines and asparagine residues, which play essential roles in this process, was reported. HIF-prolyl hydroxylases (PHs) constitute the core molecular mechanism for detecting a decrease in the partial pressure of oxygen, or hypoxia, in the cells, and are known as oxygen sensors [23,24,25,26]. In this review, I discuss the process of the molecular cloning of HIF and HIF-PHs, which explains hypoxia-induced Epo expression, the development of HIF-PH inhibitors that artificially or exogenously activate HIF by inhibiting HIF-PH, and the significance and implications of medical intervention using HIF-PH inhibitors [15].

We hope that this Special Issue will reflect the current exciting researches concerning HIFs and their applications in medicine and health science.
